# Association of ABC gene profiles with time to progression and resistance in ovarian cancer revealed by bioinformatics analyses

**DOI:** 10.1002/cam4.1964

**Published:** 2019-01-22

**Authors:** Karolina Seborova, Radka Vaclavikova, Pavel Soucek, Katerina Elsnerova, Alena Bartakova, Petr Cernaj, Jiri Bouda, Lukas Rob, Martin Hruda, Pavel Dvorak

**Affiliations:** ^1^ Toxicogenomics Unit National Institute of Public Health Prague Czech Republic; ^2^ Biomedical Center, Faculty of Medicine in Pilsen Charles University Pilsen Czech Republic; ^3^ Third Faculty of Medicine Charles University Prague Czech Republic; ^4^ Department of Gynecology and Obstetrics, Faculty of Medicine and University Hospital in Pilsen Charles University Pilsen Czech Republic; ^5^ Department of Gynecology and Obstetrics, Third Faculty of Medicine and Faculty Hospital Kralovske Vinohrady Charles University Prague Czech Republic; ^6^ Department of Biology, Faculty of Medicine in Pilsen Charles University Pilsen Czech Republic

**Keywords:** ABC transporters, bioinformatics, ovarian cancer, resistance, signatures

## Abstract

**Introduction:**

Ovarian cancer (OC) represents a serious disease with high mortality and lack of efficient predictive and prognostic biomarkers. ATP‐binding cassette (ABC) proteins constitute a large family dedicated to active transmembrane transport including transport of xenobiotics.

**Materials and methods:**

mRNA level was measured by quantitative RT‐PCR in tumor tissues from OC patients. Bioinformatics analyses were applied to two gene expression datasets (60 primary tumors and 29 peritoneal metastases). Two different approaches of expression data normalization were applied in parallel, and their results were compared. Data from publically available cancer datasets were checked to further validate our conclusions.

**Results:**

The results showed significant connections between ABC gene expression profiles and time to progression (TTP), chemotherapy resistance, and metastatic progression in OC. Two consensus ABC gene profiles with clinical meaning were documented. (a) Downregulation of *ABCC4, ABCC10, ABCD3, ABCE1, ABCF1, ABCF2,* and *ABCF3 *was connected with the best sensitivity to chemotherapy and TTP. (b) Oppositely, downregulation of *ABCB11* and upregulation of *ABCB1* and *ABCG2* were connected with the worst sensitivity to chemotherapy and TTP. Results from publicly available online databases supported our conclusions.

**Conclusion:**

This study stressed the connection between two well‐documented ABC genes and clinicopathological features—*ABCB1* and *ABCG2*. Moreover, we showed a comparable connection also for several other ABC genes—*ABCB11, ABCC4, ABCC10, ABCD3,* *ABCE1*, *ABCF1, ABCF2,* and *ABCF3*. Our results add new clinically relevant information to this oncology field and can stimulate further exploration.

## 1 INTRODUCTION

Ovarian cancer (OC) represents a heterogeneous disease with high mortality and is reported as one of the most common causes of female cancer death. A worldwide age‐standardized incidence of new cases was 6.1 and mortality 3.8 for 100 000 women in 2012.^1^ In line with the increasing trend, 22 450 new OC cases and 14 080 deaths were estimated in the USA for 2017.^2^ The risk of OC development was shown to be increased by age during menopause or menarche, abnormalities in ovulation, endometriosis, obesity, smoking, and hormonal therapy. On the other hand, factors reducing the risk of OC were reported—breast‐feeding, pregnancy, and contraception.^3^ The last update of OC classification made by WHO was released in 2014. Three main types were recognized according to the origin of tumors: epithelial, sex cord‐stromal, and germ cell tumors.^4^ Epithelial tumors represent the most common type with high‐grade serous carcinoma (HGSC) being the most frequent subtype covering more than 70% of OC cases.^5^

Standard therapy of OC is still composed of surgical treatment and/or chemotherapy nowadays. Common chemotherapy used in OC treatment is formed by a combination of platinum derivatives (carboplatin, cisplatin) and taxanes (paclitaxel, docetaxel).^6^ In recent years, therapeutic regimens based on angiogenesis inhibitors (bevacizumab, trebananib, aflibercept, pazopanib, nintedanib) were developed and entered into clinical trials. Treatment of OC patients who carry a *BRCA1/BRCA2* gene mutation can be named as an example of precision medicine approach, PARP inhibitors such as olaparib, niraparib, or rucaparib can be applied with a significant effect in this subgroup.^6^, ^7^

Despite the considerable advances in OC therapy, development of chemotherapy resistance still remains a high priority complication.Generally, the underlying mechanisms of chemoresistance are based on changes in pharmacokinetics and cytokinetics. Decreased drug activation or increased degradation stay on one side and changes in drug intake into the cell or efflux (attributed to transmembrane transporters), increased DNA repair and apoptotic pathway disturbance on the other. Several distinct mechanisms involved in these processes were addressed elsewhere, for example, the role of enzymes from cytochrome P450 family and glutathione *S*‐transferase, methyltransferase, UDP‐glucuronosyl *S*‐transferase, abnormal expression of Bcl‐2 protein family, caspases, or *TP53* mutation. The central role in the development of multidrug resistance is attributed to an increased drug efflux based on ATP‐binding cassette proteins (ABC proteins) that lead to decreased accumulation as well as efficiency of drugs inside cancer cells.^8^, ^9^

The superfamily of ABC proteins constitutes one of the biggest families dedicated to active transmembrane transport and is formed by 48 protein‐coding genes and several pseudogenes in humans. The family is divided into seven subfamilies (ABCA‐ABCG) according to the structure criteria. ABC proteins share a considerable homology; full transporters are usually formed by two nucleotide‐binding domains (NBD) and two transmembrane domains (TMD). Half transporters are formed by one NBD and one TMD and subsequently create homodimers or heterodimers.^10^ ATP hydrolysis is an essential source of energy for their active transport abilities. ABC transporters, localized to plasma membranes, membranes of mitochondria, endoplasmic reticulum, and peroxisomes, translocate a wide range of substrates—amino acids, sugars, nucleosides, vitamins, metal components, peptides, lipids, oligonucleotides, polysaccharides, and also different types of xenobiotics.^10^, ^11^

High expression of*ABCB1*, *ABCC1,* and *ABCG2* at mRNA as well as protein level was connected with the development of multidrug resistance (MDR) in a range of studies.^10^, ^11^ High expression of *ABCB1* was observed in paclitaxel and doxorubicin‐resistant cancer cell lines^12^, ^13^ and tumor tissues.^14^ For OC, overexpression of ABCB1 (at mRNA and protein level) was related to the resistance to conventional therapy based on paclitaxel, worsened outcome,^14^, ^15^, ^16^ short survival,^17^, ^18^ and treatment response.^19^, ^20^, ^21^ Association of *ABCC1* overexpression with chemoresistance, high‐grade OC, and shorter progression‐free survival (PFS) was described.^18^, ^22^, ^23^, ^24^, ^25^ *ABCG2* is known as one of the most studied MDR‐related genes, especially in the field of breast cancer. For OC, *ABCG2* overexpression is probably linked to cisplatin and paclitaxel resistance^26^ and its value as prognostic factor was shown.^27^, ^28^ Interestingly, high *CFTR* (cystic fibrosis transmembrane conductance regulator, responsible for congenital cystic fibrosis) expression was observed in advanced stages and grades of OC together with higher levels of CA‐125 and serous type occurrence. RNA interference targeted on *CFTR* resulted in decreased activity of cancer cells in vitro and in vivo in mouse xenograft models.^29^

There is still a lack of information about the other ABC genes and their role in different cancer types including OC. The main aim of the current study was to reveal new prognostic or predictive markers within ABC gene family. Bioinformatics analyses of ABC gene expression data obtained from OC samples, covering all ABC subfamilies, were conducted.

## 2 MATERIALS AND METHODS

### 2.1 Study cohort

Our comparative bioinformatics analyses were performed on a dataset collected from samples of primary ovarian tumors and peritoneal metastases. The first dataset consisted of primary tumors from 60 Czech patients diagnosed with epithelial ovarian cancer at Motol University Hospital in Prague and University Hospital in Pilsen, both localized in the Czech Republic, between the years 2009 and 2013. This patient set, where only four patients had distant metastasis, was also included in our previous work^27^ as a Pilot cohort. The clinical parameters available for this Pilot Study were as follows: age at diagnosis, clinical stage, histological grade, tumor type, distant metastasis status, neoadjuvant chemotherapy application, adjuvant or first‐line palliative chemotherapy type, Ki‐67 marker expression (only for Motol University Hospital patients), clinical outcome, and time to progression (TTP).

The second dataset consisted of peritoneal metastases from 29 OC patients, three of these patients were follow‐ups of the first dataset. These patients were diagnosed at University Hospital in Pilsen between the years 2013 and 2016 and described in detail previously.^28^ Clinical parameters available for statistical tests were as follows: age at diagnosis, clinical stage, histological grade, tumor type, distant metastasis status, neoadjuvant chemotherapy application, adjuvant or first‐line palliative chemotherapy, clinical outcome, and time to progression (TTP). Tumor tissue specimens were taken either during primary surgery (open laparotomy primary debulking or diagnostic open laparoscopy) or interval debulking surgery and immediately frozen and stored in −80°C. Detailed patients characteristics are summarized in Table S1 in the Supplemented Information.

The two cohorts and previous studies mentioned were processed in our laboratories and performed in accordance with the Declaration of Helsinki and Uniform requirements for manuscripts submitted to biomedical journals.

### 2.2 Isolation of RNA, cDNA synthesis, and qRT‐PCR

Tissue samples collected during surgical treatment were histologically verified for the appropriate tissue type—tumor or nontumor tissue. For RNA isolation, AllPrep DNA/RNA/Protein MiniKit (Qiagen, Hildesheim, Germany) was used according to the protocol recommended. The amount of isolated RNA was measured by Quant.iT RiboGreen RNA Assay Kit (Invitrogen, Eugene, USA). RevertAid First Strand cDNA Synthesis Kit (MBI Fermentas, Vilnius, Lithuania) was applied for cDNA synthesis from 0.5 μg of total RNA. Verification of cDNA quality was performed by PCR amplification of ubiquitin C fragment. Before quantitative real‐time PCR, cDNA was preamplified; this step was described in detail in our previous works.^27^, ^28^ qRT‐PCR was performed on Viia7 RT‐PCR System (Life Technologies, Carlsbad, CA, USA), with the use of TaqMan Gene Expression Assay (Life technologies) specific for each gene. The qRT‐PCR study design adhered to the Minimum Information for Publication of Quantitative Real‐Time PCR Experiments Guidelines.^30^

### 2.3 Gene expression profiling

Gene expression of 39 ABC genes (*ABCA1, ABCA2, ABCA3, ABCA7, ABCA8, ABCA9, ABCA10, ABCA12, ABCA13, ABCB1, ABCB2, ABCB3, ABCB4, ABCB5, ABCB11, ABCC1, ABCC2, ABCC3, ABCC4, ABCC5, ABCC6, ABCC7, ABCC8, ABCC9, ABCC10, ABCC11, ABCC12, ABCD1, ABCD2, ABCD3, ABCD4, ABCE1, ABCF1, ABCF2, ABCF3, ABCG1, ABCG2, ABCG5, *and *ABCG8*) was measured as described in Elsnerova et al.^27^, ^28^ for both datasets. Data of 6 genes (*ABCB5, ABCC7, ABCC8, ABCC11, ABCC12, *and *ABCG5*) were excluded, because of their expression under the detection limit, and data of 33 ABC genes entered further bioinformatics analyses.

### 2.4 Bioinformatics and statistics

Gene expression data were independently normalized according to two principles. Firstly, normalization by a set of reference genes (*PPIA, UBC,* and *YWHAZ*) was applied in line with comparative Ct method described by Livak and Schmittgen.^31^ 2^−ΔCt^ data were clustered and presented as individual data points in heat‐maps. Hierarchical clustering with Euclidean distances and Ward method was performed. Secondly, normalization by mean of gene of interest (GOI) expression was applied. Ratio of GOI Ct values to mean value was log transformed and then clustered and visualized in a similar way to the first type of normalization.

Nonparametric statistical tests (Mann‐Whitney U and Kruskal‐Wallis tests) were used to take into account statistically significant relationships between expression data clusters and clinicopathological features. Drug resistance was evaluated as follows: Patients who presented with progression, recurrence, or death (therapy failure) in <6 months since termination of adjuvant therapy were considered resistant, those with therapy failure in the range of 6‐12 months had intermediate status, and patients in remission at 12 months or later after adjuvant therapy completion were considered sensitive.^32^ TTP was defined as time elapsed from surgical treatment to disease progression, recurrence, death from any cause, or to the last examination date without evidence of disease. Median TTP was estimated using the Kaplan‐Meier method, and the difference was tested using the log‐rank test. Two‐sided *P*‐values of <0.05 were considered statistically significant. Microsoft Excel 2013 and Statistica software (TIBCO Software Inc, Palo Alto, CA, USA) were used for preprocessing of raw expression data. Hierarchical clustering and heat‐map visualization were performed with the help of Statistica software and online freely available bioinformatics tools Heatmapper^33^ and Shinyheatmap.^34^ Statistical tests were done in the Statistica software.

Publically available web‐based tools—Kaplan‐Meier Plotter and PrognoScan,^35^, ^36^ evaluating among others data from Gene Expression Omnibus and The Cancer Genome Atlas databases, were searched owing to the effort to collect independent data supporting the prognostic significance of the ABC profiles defined in the study.

## 3 RESULTS

### 3.1 Association of ABC clusters with TTP and chemoresistance in primary OC tumors

**Figure 1:**
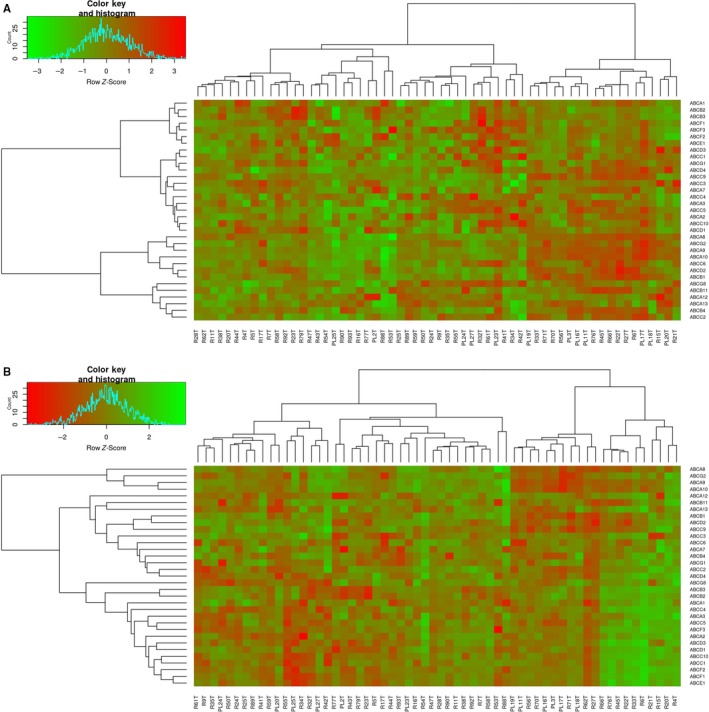
Hierarchical clustering of the primary tumor OC dataset based on 33 ABC gene expression measurements. A, Heat‐map visualization of data normalized by a set of reference genes (clusters R1‐R4). B, Heat‐map visualization of data normalized by mean of gene of interest expression (clusters G1‐G4)

**Figure 2:**
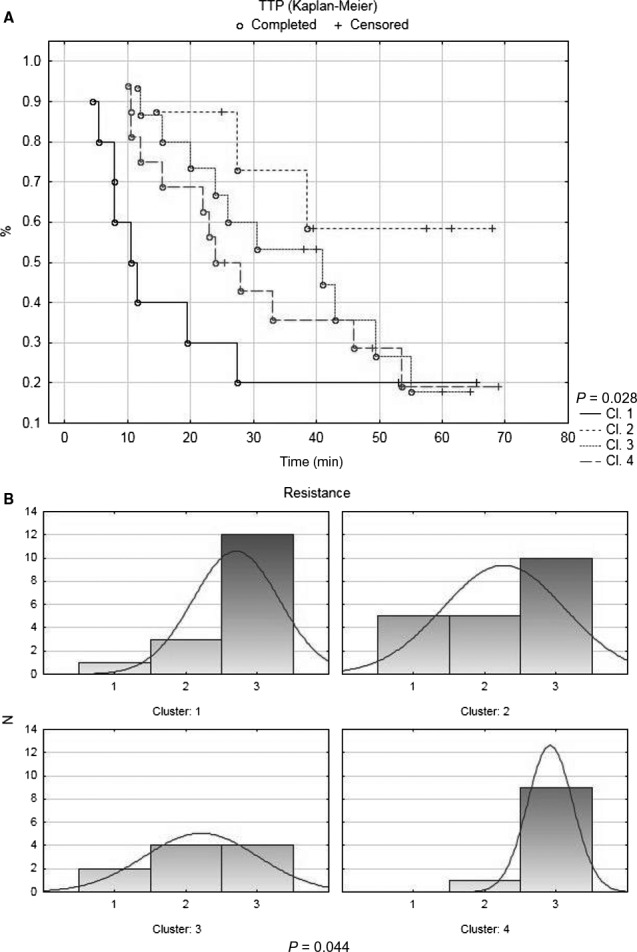
ABC gene profiles defined by this study were tested for associations with known clinicopathological features. A, Kaplan‐Meier curves based on time to progression (TTP) analysis are shown for the clusters R1‐R4. B, Histograms showing the distribution of chemotherapy‐sensitive (#3), intermediately resistant (#2), and resistant (#1) patients between the clusters G1‐G4 are presented

Hierarchical clustering of the first dataset, based on data normalized by set of reference genes, divided 60 patients into four main clusters (R1‐R4). Cluster R1 consisted of 14 patients, cluster R2 consisted of 11 patients, cluster R3 consisted of 16 patients, and cluster R4 consisted of 19 patients. Heat‐map visualization of the clusters R1‐R4 is shown in Figure 1A. There were no statistically significant differences in age, clinical stage, histological grade, tumor type, distant metastasis status, chemotherapy administration, and resistance to treatment between these clusters. However, TTP analysis revealed significant differences in this parameter (*P* = 0.028). Median TTP for cluster R1 was 10 months, for R2 median TTP was not reached, for R3 was 41 months, and for R4 24 months. Kaplan‐Meier curves based on TTP analysis are shown in Figure 2A. The cluster R1 with the worst TTP was characterized by an ABC profile where *ABCA1, B2, B3, C3, D1,* and *G1* were upregulated and *ABCA12, B11,* and *G8* were downregulated. Oppositely, the cluster R2 with the best TTP was defined by downregulation of *ABCA8, A9, A10, B1, B4, B11, C2, C5, C6, D2, D4, F3, G1, G2,* and *G8*, and no gene was upregulated in this cluster. The comparison between the characteristics of the most important ABC profiles recognized in this study is presented in Table 1.

**Table 1:** Comparison between the most important ABC gene profiles recognized by this study

Genes	Clusters						
	Primary tumors				Peritoneal metastases		Merged dataset
	Clustering based on normalization to REF genes		Clustering based on normalization by mean expression of GOI		Clustering based on normalization by mean expression of GOI^a^		Clustering based on normalization to REF genes^b^
	R1	R2	G3	G4	SG1	SG2	MR5
	Worst TTP	Best TTP	Worst sensitivity	Best sensitivity	Worst TTP, sensitivity	Best TTP, sensitivity	Metastases, worst TTP
ABCA1	U	I	I	D	I	I	I
ABCA2	I	I	I	D	I	I	I
ABCA3	I	I	I	D	I	I	I
ABCA7	I	I	I	D	I	I	I
ABCA8	I	D	U	I	D	U	U
ABCA9	I	D	U	I	D	U	U
ABCA10	I	D	U	I	D	U	U
ABCA12	D	I	D	D	I	I	I
ABCA13	I	I	I	D	I	I	I
ABCB1	I	D	U	I	I	I	U
ABCB2	U	I	I	D	I	I	I
ABCB3	U	I	I	D	I	I	I
ABCB4	I	D	I	D	I	I	U
ABCB11	D	D	I	I	D	U	I
ABCC1	I	I	I	D	I	I	I
ABCC2	I	D	I	D	I	I	U
ABCC3	U	I	I	D	I	I	I
ABCC4	I	I	I	D	U	D	I
ABCC5	I	D	I	D	I	I	I
ABCC6	I	D	I	D	I	I	U
ABCC9	I	I	U	I	D	U	U
ABCC10	I	I	I	D	U	D	I
ABCD1	U	I	I	D	I	I	I
ABCD2	I	D	U	I	D	U	U
ABCD3	I	I	I	D	U	D	I
ABCD4	I	D	I	D	I	I	U
ABCE1	I	I	I	D	U	D	I
ABCF1	I	I	I	D	U	D	I
ABCF2	I	I	I	D	U	D	I
ABCF3	I	D	I	D	U	D	I
ABCG1	U	D	I	D	I	I	I
ABCG2	I	D	U	D	I	I	U
ABCG8	D	D	I	D	I	I	I

D, downregulation; I, intermediate expression; TTP, time to progression; U, upregulation.

a Clustering based on normalization to REF genes—did not reveal any significant expression changes.

b Clustering based on normalization to GOI mean—did not reveal any significant expression changes.

Hierarchical clustering of the first dataset, based on data normalization by mean expression of GOI, divided 60 patients also into four main clusters (G1‐G4). Cluster G1 consisted of 17 patients, cluster G2 consisted of 22 patients, cluster G3 consisted of 11 patients, and cluster G4 consisted of 10 patients. Heat‐map visualization of the clusters G1‐G4 is shown in Figure 1B. There were no statistically significant differences in age, clinical stage, tumor type, distant metastasis status, and TTP between these clusters. However, we revealed significant differences in histological grade distribution (*P* = 0.017) and chemotherapy resistance status (*P* = 0.044). The ratios of Grade 1 and 2 patients to all patients within the clusters were as follows: 8/17 (47%) for G1, 2/21 (10%) for G2, 1/11 (9%) for G3, and 4/10 (40%) for G4. The ratio of chemotherapy‐sensitive patients to all patients was for the cluster G1 12/16 (75%), for G2 10/20 (50%), for G3 4/10 (40%), and for G4 9/10 (90%). The order of clusters according to the increasing ratio of chemotherapy‐sensitive patients was as follows: G3, as the worst cluster from this point of view, then G2, G1, and G4, as the cluster with the best performance. Histograms showing the distribution of chemotherapy‐sensitive, intermediately resistant, and resistant patients within the clusters are disclosed in Figure 2B. The cluster G3 with the largest proportion of resistant tumors was characterized by an ABC profile where *ABCA8, A9, A10, B1, C9, D2,* and *G2* were upregulated and only *ABCA12* was downregulated. Oppositely, the cluster G4 with the largest proportion of sensitive tumors was characterized by downregulation of all the ABC genes tested with the exception of *ABCA8, A9, A10, B1, B11, C9,* and *D2*, which all had intermediate level of expression. Clusters G3 and G4 were included into the comparison presented in Table 1.

### 3.2 Association of ABC clusters with TTP and chemoresistance in OC peritoneal metastases

Hierarchical clustering of the second dataset, based on data normalized by set of reference genes, divided 29 patients into three main clusters (SR1‐SR3). Clusters consisted of 11 (SR1), 8 (SR2), and 10 patients (SR3). Heat‐map visualization of the clusters is shown in Figure S1A in the Supplemented Information. There was no statistically significant difference between these main clusters for any of the clinicopathological features tested.

Hierarchical clustering of the second dataset, based on data normalization by mean expression of GOI, divided 29 patients also into three main clusters (SG1‐SG3). Clusters consisted of 17 (SG1), 8 (SG2), and 4 patients (SG3). Heat‐map visualization of these clusters is shown in Figure S1B in the Supplemented Information. There were no statistically significant differences in age, clinical stage, histological grade, tumor type, and chemotherapy administration. However, there were significant differences in TTP (*P* = 0.027) and chemotherapy resistance (*P* = 0.024). Median TTP for cluster SG1 was 11 months, for SG2 27 months, and for SG3 13 months. Kaplan‐Meier curves based on TTP analysis are shown in Figure S2A. The ratio of chemotherapy‐sensitive patients to all patients was for the cluster SG1 2/11 (18%), for SG2 4/5 (80%), and for SG3 1/3 (33%). Histograms showing the distribution of chemotherapy‐sensitive, intermediate resistant, and resistant patients are disclosed in Figure S2B. The cluster SG1 with the worst TTP and highest proportion of resistant tumors was defined by the upregulation of *ABCC4, C10, D3, E1, F1, F*2, and *F3*, and the downregulation of *ABCA8, A9, A10, B11, C9,* and *D2*. Oppositely, the cluster SG2 with the best TTP and highest proportion of sensitive tumors was characterized by an adverse ABC profile—upregulation of *ABCA8, A9, A10, B11, C9,* and *D2* and downregulation of *ABCC4, C10, D3, E1, F1, F2,* and *F3*. These two clusters were also included into the comparison presented in Table 1.

### 3.3 Association of TTP with ABC clusters in the merged dataset

Data from first (primary tumors) and second (peritoneal metastases) datasets were merged, and hierarchical clustering followed by statistical analyses was performed on the merged dataset in a similar way as described above. Based on data normalization by reference genes, 89 patients were sorted into five main clusters (MR1‐MR5). The number of patients assigned to these clusters was as follows: 17 in MR1, 14 in MR2, 5 in MR3, 27 in MR4, and 26 in MR5. The relevant heat‐map can be viewed in Figure S3A in the Supplemented Information. There were no statistically significant differences in age, clinical stage, tumor type, and chemotherapy resistance status. Analyses showed significant differences in histological grade (*P* = 0.026) and TTP (*P* = 0.049). The ratio of Grade 1 and 2 patients to all patients was 6/17 (35%) in MR1, 1/13 (8%) in MR2, 3/5 (60%) in MR3, 3/24 (13%) in MR4, and 3/23 (13%) in MR5. The median TTP (in the increasing order) was as follows: 16 months for MR4, 20 for MR5, 28 for MR2, and 42 for MR1 and was not reached for MR3. However, the cluster MR5 started to have the worst TTP after 33 months of follow‐up. Kaplan‐Meier curves derived from this TTP analysis are shown in Figure S3B. The cluster MR5 with the highest ratio of patients from the peritoneal metastases set and the worst TTP could be characterized by upregulation of *ABCA8, A9, A10, B1, B4, C2, C6, C9, D2, D4,* and *G2* (Table 1). Oppositely, the cluster MR3 with the best TTP could be characterized by the downregulation of these 11 ABC genes.

Hierarchical clustering after data normalization by GOI mean expression separated patients in the merged dataset also into five main clusters; however, no significant differences in clinical data were found between them.

### 3.4 Results from publicly available online databases supported our conclusions

Analysis based on data from Kaplan‐Meier Plotter database showed better progression‐free survival (PFS), with statistical significance *P* = 0.0025, for OC patients displaying low expression of the ABC profile—*ABCC4, ABCC10, ABCD3, ABCE1, ABCF1, ABCF2*, and *ABCF3*. Cohort of 614 OC patients was evaluated for this analysis (Kaplan‐Meier curves are disclosed in Figure S4A). Evaluation of the significance of *ABCB11* expression ended with significantly (*P* = 0.029) worse PFS for patients with low expression of this gene; this is also in agreement with our results. Cohort of 1435 OC patients were tested in case of *ABCB11* (Figure S4B). Regarding *ABCB1* and *ABCG2* genes, the test on 1435 OC patients showed significantly better PFS for the first 60 months in those with high expression of these two genes, after this time point, the curves for high and low expression were identical (Figure S4C). The third analysis in the Kaplan‐Meier Plotter did not confirm our conclusion regarding *ABCB1* and *ABCG2* genes.

We further evaluated the prognostic significance of *ABCB1* and *ABCG2* genes individually in PrognoScan database, because this web‐tool does not offer evaluation of gene profiles. Regarding *ABCB1* gene in OC studies, 2 out of 6 datasets showed statistically significant results—DUKE‐OC and GSE26712. Patients with high expression showed worse OS in both datasets (*P* = 0.010 and *P* = 0.034). Regarding *ABCG2* gene in OC studies, 1 out of 6 datasets showed significant values–GSE26712. Patients with high expression were found to have worse disease‐free survival (DFS) as well as OS (*P* = 0.007 and *P* = 0.002).

## 4 DISCUSSION

In the current study, we were inspired by the lack of efficient prognostic or predictive biomarkers in OC, which represents one of the most serious cancer types. We evaluated datasets based on mRNA level measurements by qRT‐PCR in tissue samples from primary tumors and peritoneal metastases obtained during surgical treatment of OC patients. Expression levels of a set of 33 genes from the ABC family, covering all 7 subfamilies, were above the detection limit and evaluated in our bioinformatics analyses. Two different approaches of expression data normalization were applied, and the results were compared.

Previously, ABC gene expression profiles were connected with stem cell pluripotency in the work conducted by Barbet et al.^37^ Other studies found associations of ABC profiles with pathologic response to chemotherapy in breast cancer patients^38^ or chemoresistance in acute myeloid leukemia.^39^ Two previous works of our team focused on ABC profiles. We reported a connection between ABC profiles from tumor tissues and several clinicopathological features such as DFS in breast cancer, regional lymph node metastasis (pN) in colorectal cancer, and primary tumor size (pT), histological grade and clinical stage in a merged breast, colorectal and pancreatic cancer dataset.^40^ We also demonstrated a compelling correlation between ABC profiles from nontumor tissues of cancer patients and distant metastasis status (cM) and overall survival (OS) in colorectal and pancreatic cancers.^41^

The results of the current study on OC patients showed notable connections between ABC gene expression profiles and TTP, chemotherapy resistance, and metastatic progression. Importantly, we found two consensus ABC gene profiles with clinical meaning. Downregulation of *ABCC4, ABCC10, ABCD3, ABCE1, ABCF1, ABCF2,* and *ABCF3 *was connected with positive features—the best sensitivity to chemotherapy and TTP. Oppositely, downregulation of *ABCB11* and upregulation of *ABCB1* and *ABCG2* were connected with negative features—the worst sensitivity to chemotherapy and TTP (summarized in Table 1).

Thus, this study shows for the first time that clusters composed of several drug resistance connected ABCs (and in some cases also ABCs without known drug translocating activity) may predict drug sensitivity and prognosis including metastatic potential of OC tumors. This approach, if confirmed by independent follow‐up studies, may prove to be more vital than the concept of single gene or ABC subfamily protein studies in cancer prognosis and drug response prediction. Moreover, the prognostic expression profile may be more general as we recently demonstrated in pancreatic and colorectal carcinomas.^40^, ^41^

The results of several genes—particularly*ABCA8, ABCA9, ABCA10, ABCB11, ABCC9*, and *ABCD2*—were discordant between our two patient sets. Downregulation and upregulation of these genes were associated with positive and negative features, respectively, in primary tumors; however, the situation was reversed in peritoneal metastasis. This could be attributed to the nondirect relationships between these genes and features tested as well as the clinical differences between the two patient sets. Notably, these results support the conclusion of our previous studies,^27^, ^28^ where especially the role of ABCA9, ABCA10, and ABCC9 transporters in OC progression and metastatic spread was stressed. Connection between high expression of ABC transporters from the subfamily A and poor outcome in serous OC was recently discussed in the study conducted by Hedditch et al.^42^ Furthermore, the relatively small number of patients and the differences between clinical characteristics of both OC patient cohorts may be seen as limitations of the current study. However, the study was designed as a pilot study preceding more robust validation in larger patient sets.

The present study has also shown an innovative approach in terms of methodology—combination of two data normalization procedures. Both approaches applied for expression data normalization have their advantages and disadvantages. The normalization by a set of reference genes represents the most used and evaluated approach. This solution can reduce the effect of intersample and interexperiment variation; however, it is based on the prerequisite that reference genes have a stable expression in all samples during the whole experiment, a condition which should be tested prior a study opening. The normalization by mean of GOI expression does not depend on the stability of reference genes; however, it is based on the prerequisite that there were the same amount and quality of starting RNA in all samples. The qualitative condition is hard to achieve in clinical specimens with different handling in terms of time since specimens removal from patient to its storage. Combination of the two approaches of data normalization and comparison of results in independent patient sets represent, according to our opinion, an efficient way of robust result evaluation.

There is some evidence about the role of ABC genes other than the most studied ones in OC, which we would like to mention in the following paragraphs. Most information on proteins from ABC superfamily was published for ABCB1, ABCC1, and ABCG2. An increased ABCB1 (mRNA and protein level) expression in association with resistance to paclitaxel was observed in vitro and in tumor tissue.^12^, ^43^ In the present study, the treatment of patients was based on paclitaxel. ABCB1 upregulation was observed in OC tumor tissue^15^ and ABCB1 level associated with higher risk of progression of the disease and also with poor response to the treatment,^16^, ^19^, ^21^ but not with OS.^16^ The above published data support the connection of *ABCB1* upregulation with poor TTP and chemotherapy resistance in the present study. Congruently, ABCB1 upregulation in OC patients with shorter PFS was also observed.^18^ ABCB11, which shares considerable structural homology with ABCB1, was associated with resistance to paclitaxel after transfection of *ABCB11* into ovarian cancer cells.^44^

Although ABCC family of multidrug resistance proteins belongs to a more frequently studied protein groups, besides ABCC1 and ABCC2, the other members are much less recognized and very rarely studied in OC. Connection between ABCC4 upregulation and shorter PFS in OC patients^23^ and correlation between drug resistance and *ABCC9* gene amplification in vitro^45^ were reported.

*ABCD2*, encoding transporter located on the peroxisome membrane,^46^ was associated with resistance to platinum derivatives and *ABCD2* knockdown increased apoptosis in the SKOV3 OC model cell line following cisplatin treatment.^47^ The present study results also confirm a potential role of this gene in the resistance to platinum‐based treatment in OC patients.

Unlike the vast majority of members of the ABC family, members of the ABCF and ABCE families are not membrane transporters, but are involved in initiation phase of translation.^48^, ^49^ *ABCF2* expression appears to be affected by treatment as higher expression in the post‐treatment group of HGSC patients compared to paired pretreatment samples was observed.^50^ Higher ABCF2 protein expression in patients not responding to treatment with platinum‐based chemotherapy in clear cell ovarian carcinoma was reported,^51^ and higher *ABCF2* expression was observed in cisplatin‐resistant OC A2780 cell subline.^52^ Thus, the published facts are in concordance with our results, which describe a connection between downregulation of all ABCF family members with better chemotherapy resistance and TTP.

*ABCG2* gene is associated with multidrug resistance, especially in breast cancer. *ABCG2* overexpression in vitro associates with increased resistance to paclitaxel and cisplatin, a drug combination modality commonly used in OC treatment.^26^ In the previous study, this gene was suggested as the most interesting prognostic marker for OC,^27^ and in the present study, its upregulation was observed within the MR5 cluster of genes associated with the presence of metastases and poor TTP of the merged set of all patients.

Transporters ABCC10, D3, E1, F1, and F3 belong to the less studied ones, and besides gene expression deregulation in diverse tumors, no significant relevance to clinical‐pathological parameters was published. Thus, these study results show, for a first time, their significant relationships with chemotherapy resistance and TTP of OC patients.

In conclusion, this study stressed the connection between two well‐documented ABC genes and clinicopathological features—upregulation of *ABCB1* and *ABCG2* correlated with worse sensitivity to chemotherapy and worse TTP. Moreover, we showed a comparable connection also for several other ABC genes—downregulation of *ABCB11* also correlated with worse sensitivity to chemotherapy and worse TTP, and oppositely, downregulation of *ABCC4, ABCC10, ABCD3, ABCE1, ABCF1, ABCF2,* and *ABCF3 *correlated with better sensitivity to chemotherapy and better TTP. These results were supported by the searches in the two publically available cancer databases (Kaplan‐Meier Plotter and PrognoScan), however, only partly in case of the genes *ABCB1* and *ABCG2*. The study brings new clinically relevant information in the ovarian cancer field and has the potential to stimulate further exploration.

## CONFLICT OF INTEREST

The authors have no relevant affiliations or financial involvement with any organization or entity with a financial interest in or financial conflict with the subject matter or materials discussed in the manuscript. This includes employment, consultancies, honoraria, stock ownership or options, expert testimony, grants or patents received or pending, or royalties.
