# Supplementary figures and images for: Association of ABC gene profiles with time to progression and resistance in ovarian cancer revealed by bioinformatics analyses
Source: Cancer Med. 2019 Jan 22;8(2):606–16. doi: 10.1002/cam4.1964 (PMC6382717; doi:10.1002/cam4.1964)

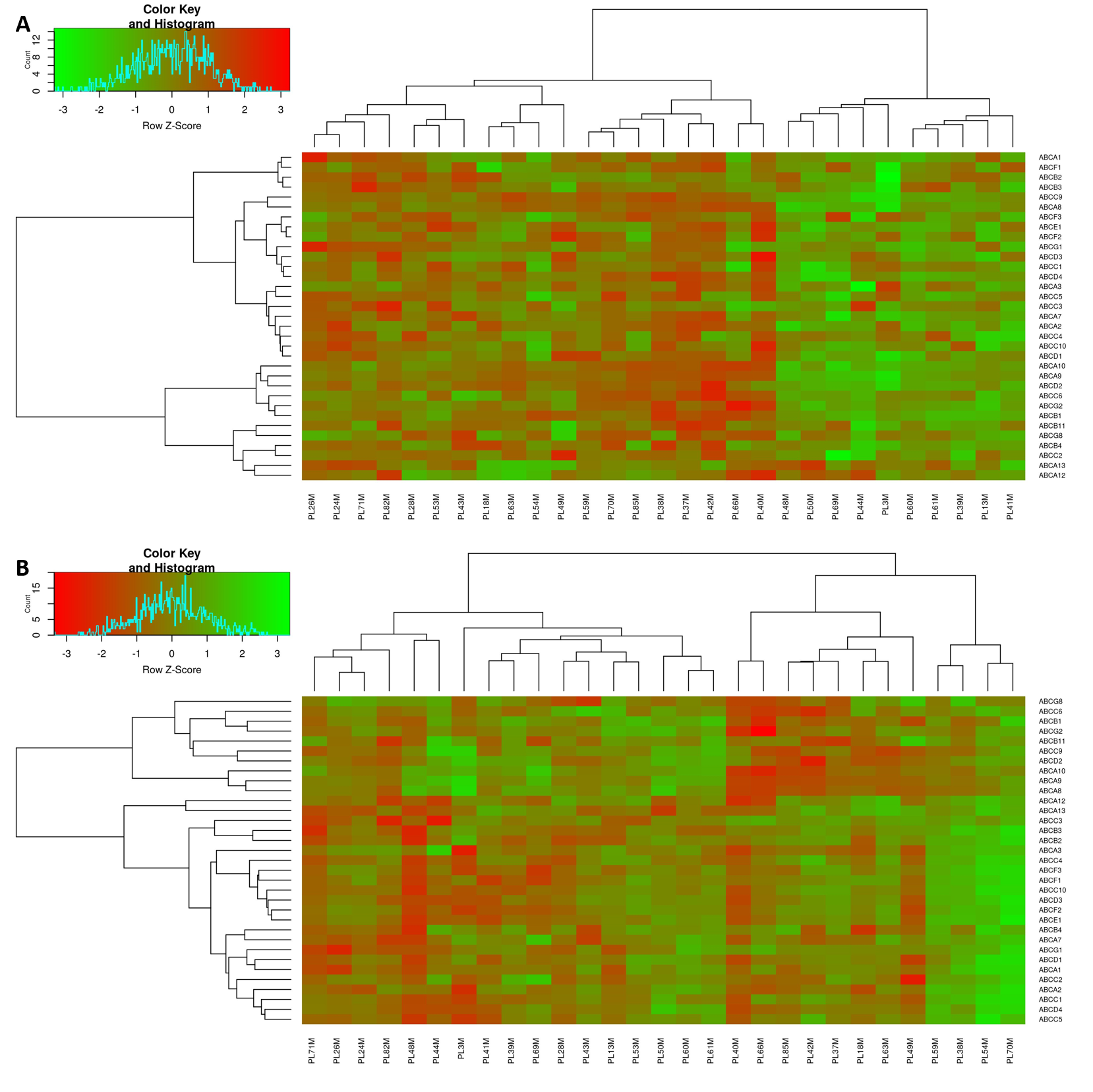

Supplement: Supplementary file 1 [file CAM4-8-606-s001.tif]

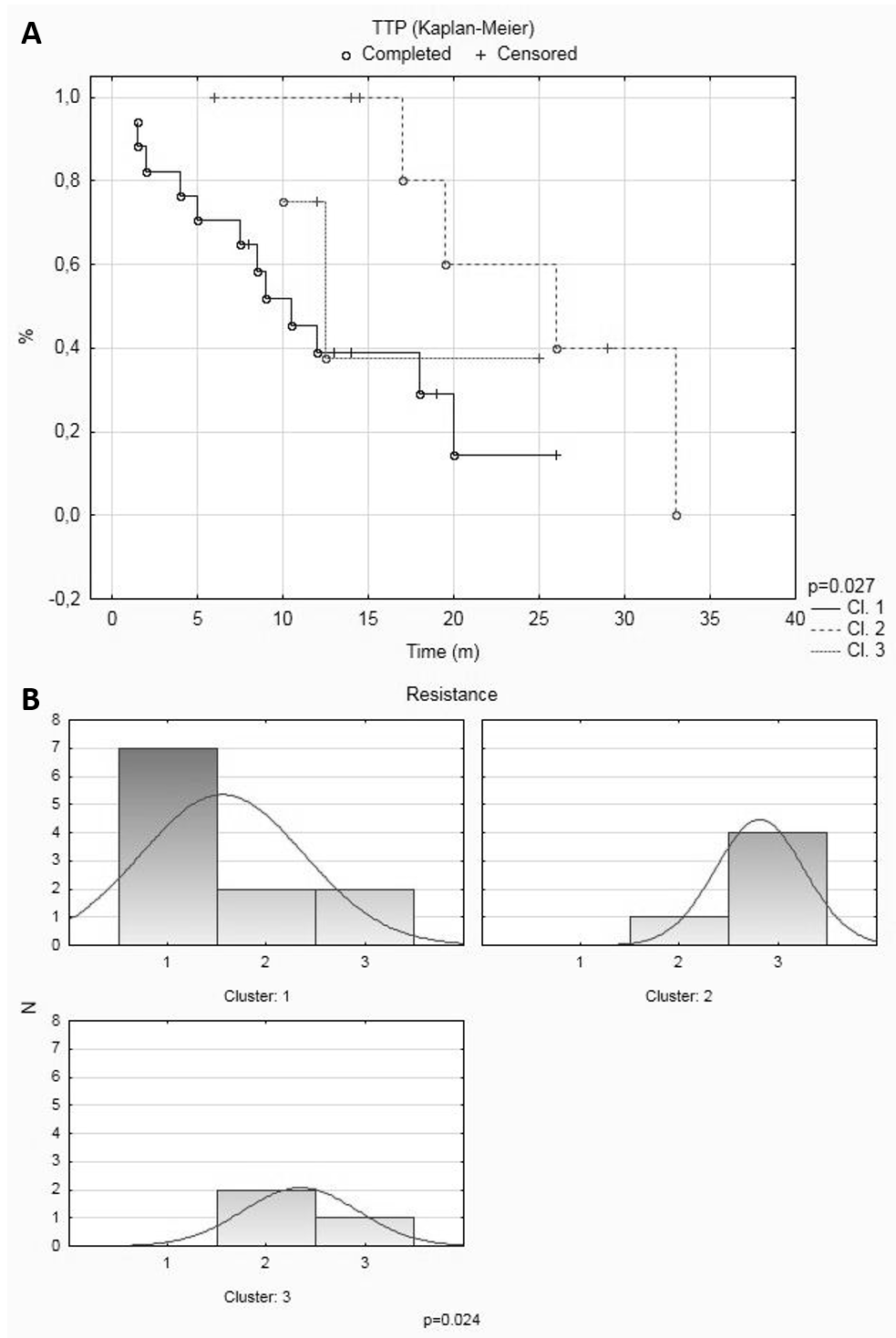

Supplement: Supplementary file 2 [file CAM4-8-606-s002.tif]

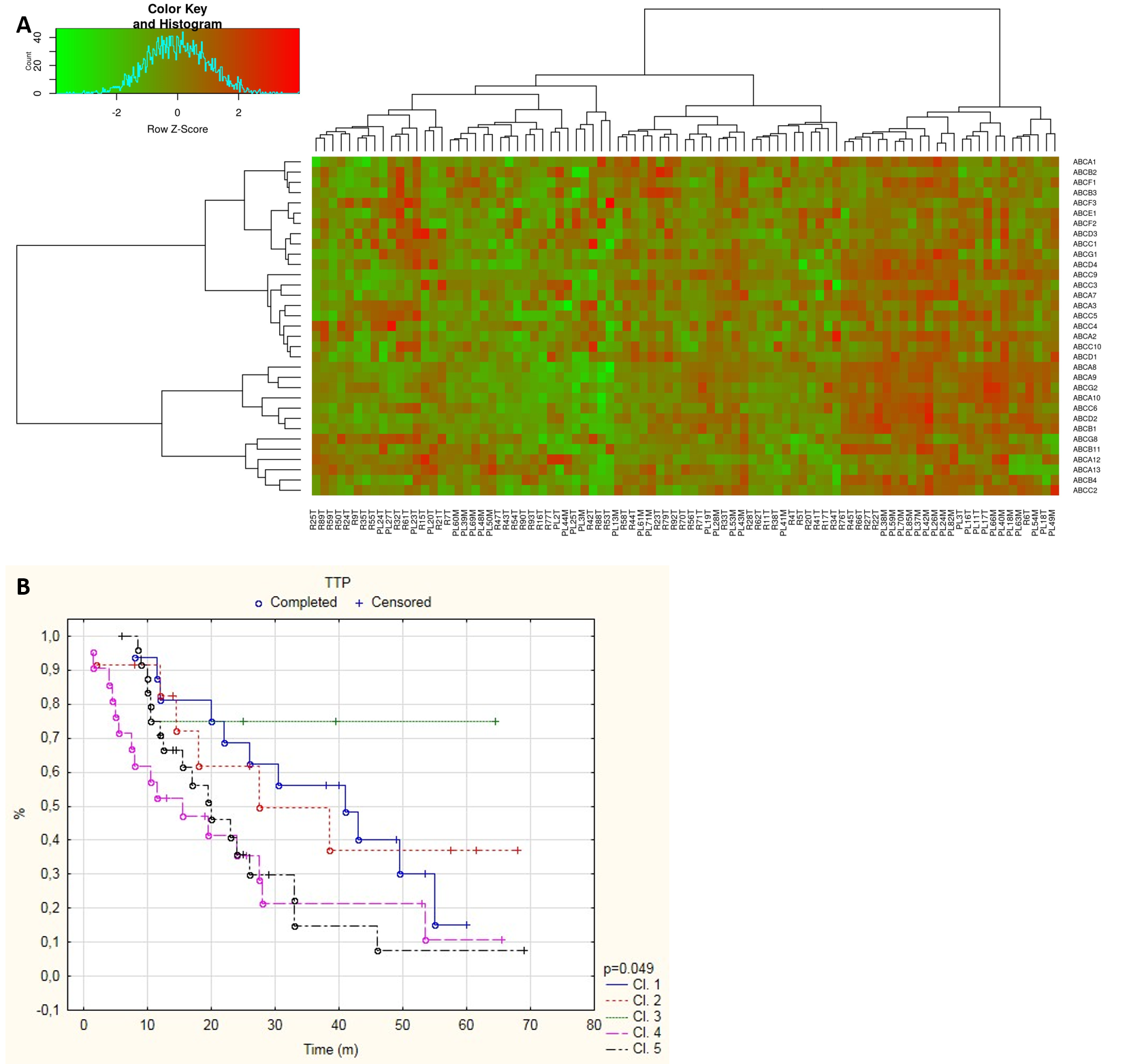

Supplement: Supplementary file 3 [file CAM4-8-606-s003.tif]

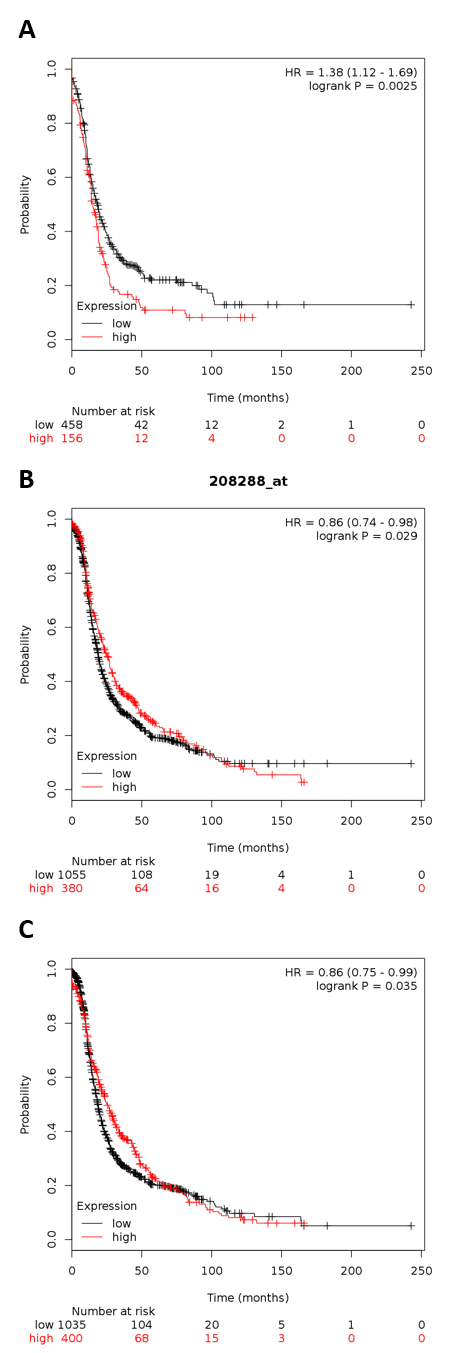

Supplement: Supplementary file 4 [file CAM4-8-606-s004.tif]
