# Supplementary material for: Association of ABC gene profiles with time to progression and resistance in ovarian cancer revealed by bioinformatics analyses
Source: Cancer Med. 2019 Jan 22;8(2):606–16. doi: 10.1002/cam4.1964 (PMC6382717; doi:10.1002/cam4.1964)
Supplement: Supplementary file 5 [file CAM4-8-606-s005.docx]

| Table S1: Detailed patient characteristics of the two datasets compared in the study | | | | |
| --- | --- | --- | --- | --- |
|  | **First dataset** | **Second dataset** |  |  |
| **N** | 60 | 29 |  |  |
| **Age (median)** | 62.5 ± 11.2 | 62.0 ± 12.7 |  |  |
| **Stage** | | |  |  |
| I | 4 | 0 |  |  |
| II | 6 | 0 |  |  |
| III | 41 | 25 |  |  |
| IV | 4 | 3 |  |  |
| NA | 5 | 1 |  |  |
| **EOC type** | | |  |  |
| others | 10 | 4 |  |  |
| HGSC | 45 | 22 |  |  |
| not available | 5 | 3 |  |  |
| **Grade** | | |  |  |
| 1 | 5 | 0 |  |  |
| 2 | 11 | 1 |  |  |
| 3 | 43 | 25 |  |  |
| not available | 1 | 3 |  |  |
| **pM** | | |  |  |
| present | 4 | 3 |  |  |
| absent | 46 | 10 |  |  |
| not available | 10 | 17 |  |  |
| **Neoadjuvant chemotherapy (NACT)** | | |  |  |
| administred | 10 | 18 |  |  |
| not administred | 50 | 11 |  |  |
| **Regimen of NACT** | | |  |  |
| paclitaxel monotherapy | 1 | 0 |  |  |
| paclitaxel with carboplatin | 5 | 14 |  |  |
| platinum monotherapy | 2 | 0 |  |  |
| other regimens | 1 | 4 |  |  |
| not available | 1 | 0 |  |  |
| **Adjuvant chemotherapy** | | |  |  |
| administred | 60 | 27 |  |  |
| not administred | 0 | 2 |  |  |
| **Regimen of adjuvant chemotherapy** |  |  |  |  |
| paclitaxel monotherapy | 1 | 0 |  |  |
| paclitaxel with carboplatin | 32 | 17 |  |  |
| paclitaxel with cisplatin | 5 | 0 |  |  |
| paclitaxel with carboplatin/cisplatin | 15 | 0 |  |  |
| platinum monotherapy | 4 | 2 |  |  |
| other regimens | 2 | 8 |  |  |
| not available | 1 | 0 |  |  |
